# Novel *LYST* Variants Lead to Aberrant Splicing in a Patient with Chediak–Higashi Syndrome

**DOI:** 10.3390/genes16010018

**Published:** 2024-12-26

**Authors:** Maxim Aleksenko, Elena Vlasova, Amina Kieva, Ruslan Abasov, Yulia Rodina, Michael Maschan, Anna Shcherbina, Elena Raykina

**Affiliations:** 1 Dmitry Rogachev National Medical Center of Pediatric Hematology, Oncology and Immunology, 117198 Moscow, Russiaelena.raykina@dgoi.ru (E.R.); 2 Regional Children’s Clinical Hospital, 620102 Yekaterinburg, Russia

**Keywords:** Chediak–Higashi syndrome, *LYST*, immunodeficiency, hypopigmentation, next-generation sequencing, in silico splice site prediction tools, SpliceAI, aberrant splicing, mRNA (cDNA) sequencing

## Abstract

**Background:** The advent of next-generation sequencing (NGS) has revolutionized the analysis of genetic data, enabling rapid identification of pathogenic variants in patients with inborn errors of immunity (IEI). Sometimes, the use of NGS-based technologies is associated with challenges in the evaluation of the clinical significance of novel genetic variants. **Methods:** In silico prediction tools, such as SpliceAI neural network, are often used as a first-tier approach for the primary examination of genetic variants of uncertain clinical significance. Such tools allow us to parse through genetic data and emphasize potential splice-altering variants. Further variant assessment requires precise RNA assessment by agarose gel electrophoresis and/or cDNA Sanger sequencing. **Results:** We found two novel heterozygous variants in the coding region of the *LYST* gene (c.10104G>T, c.10894A>G) in an individual with a typical clinical presentation of Chediak–Higashi syndrome (CHS). The SpliceAI neural network predicted both variants as probably splice-altering. cDNA assessment by agarose gel electrophoresis revealed the presence of abnormally shortened splicing products in each variant’s case, and cDNA Sanger sequencing demonstrated that c.10104G>T and c.10894A>G substitutions resulted in a shortening of the 44 and 49 exons by 41 and 47 bp, respectively. Both mutations probably lead to a frameshift and the formation of a premature termination codon. This, in turn, may disrupt the structure and/or function of the LYST protein. **Conclusions:** We identified two novel variants in the *LYST* gene, predicted to be deleterious by the SpliceAI neural network. Agarose gel cDNA electrophoresis and cDNA Sanger sequencing allowed us to verify inappropriate splicing patterns and establish these variants as disease-causing.

## 1. Introduction

Chediak–Higashi syndrome (OMIM #214500) is a rare autosomal recessive genetic disorder caused by biallelic mutations in the Lysosomal Trafficking Regulator gene (*LYST*, 1q42.3, OMIM *606897). This gene consists of 53 exons and encodes a large cytosolic protein of 425 kDa (3801 amino acids), which belongs to the BEACH (Beige And Chediak–Higashi) protein family [1]. The BEACH family includes various proteins that share the same C-terminal architecture consisting of three domains: a Pleckstrin-homology domain, a BEACH domain (Beige and Chediak–Higashi), and several WD40 repeats [2,3]. The LYST protein is responsible for the regulation of lysosomal trafficking and the synthesis, fusion, and transport of cytoplasmic granules [4]. Clinically, CHS is characterized by partial oculocutaneous albinism, neurodevelopmental abnormalities, and immunodeficiency of variable degree manifesting in recurrent infections, cytopenias and other autoimmune symptoms, mild bleeding tendency, and increased risk of developing hemophagocytic lymphohistiocytosis (HLH)—a hyperinflammatory condition with a potentially lethal outcome. The presence of inclusion bodies of lysosomal origin in various blood cells, which are composed of lysosomes, melanosomes, platelet dense granules, and cytolytic granules, is a pathognomonic feature of CHS [5,6]. Enlarged lysosomes were first described by Chediak in 1952 and Higashi in 1954 [7,8,9,10]. Giant intracytoplasmatic granulations are the hallmark of this disorder, displaying endosomal/lysosomal characteristics and impaired leucocyte function, leading to immune dysfunction [6,11]. According to the classification of IEI, compiled by the International Union of Immunological Societies (IUIS) [12], CHS belongs to the diseases of immune dysregulation, the subgroup of familial hemophagocytosis with hypopigmentation, which also includes Griscelli and Germansky–Pudlak syndromes. Fewer than 500 cases of CHS have been reported in the literature worldwide [6]. The treatment of CHS using hematopoietic stem cell transplantation is effective in treating hematologic and immune defects, but it does not prevent progressive neurological dysfunction [6,13]. In this study, we report two novel heterozygous variants in the coding region of the *LYST* gene in an individual with a clinical presentation of Chediak–Higashi syndrome and demonstrate an abnormal splicing signature in both variants’ cases.

## 2. Materials and Methods

### 2.1. Patient

**Figure 1:**
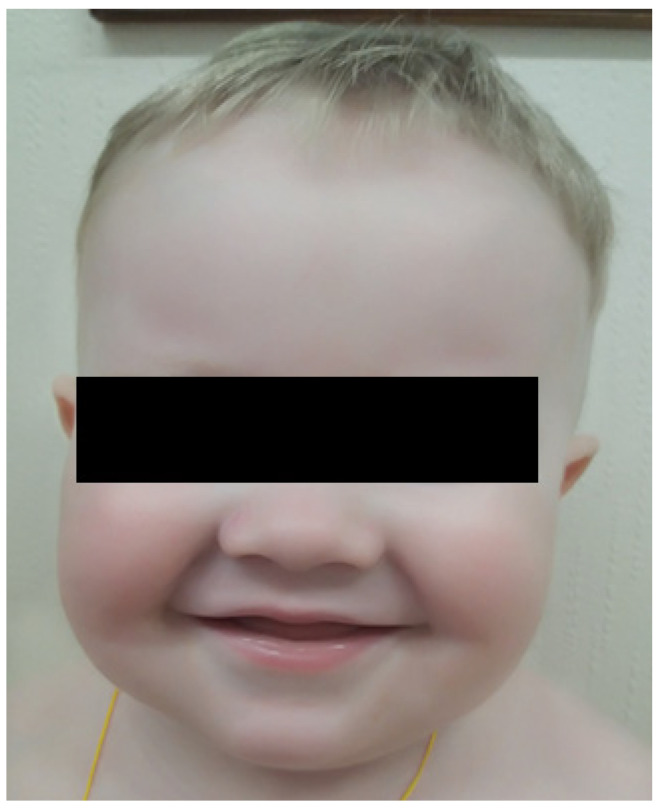
Patient’s visual appearance.

**Figure 2:**
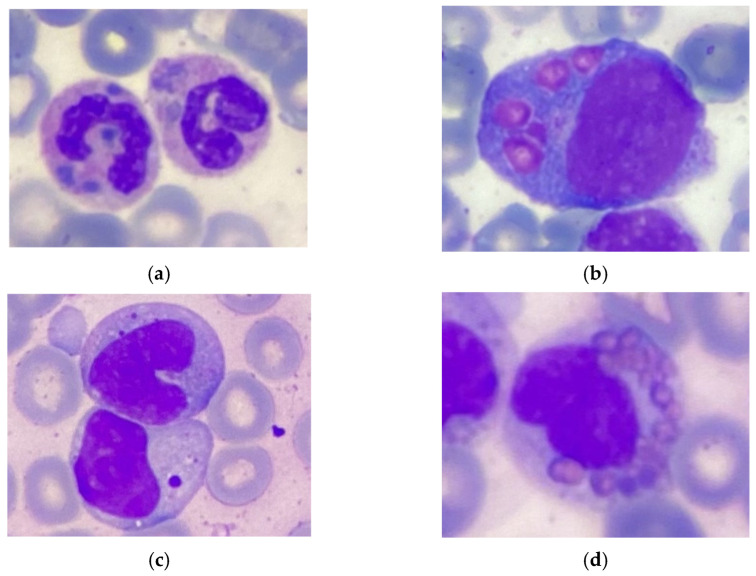
Pathognomonic giant inclusions in bone marrow cells. (**a**) Neutrophil. (**b**) Promonocyte. (**c**) Lymphocyte. (**d**) Eosinophil. Stained with hematoxylin and eosin.

Here, we report an 18-month-old male born of a non-consanguinous marriage. Both parents and the patient’s two sisters are clinically healthy, and there was no family history of immunodeficiency or hematological disorders. Shortly after birth, the patient was found to have elevated acute phase reactants, attributed to intrauterine infection of unknown etiology, neurological abnormalities, and profound thrombocytopenia (platelet count 98 × 10^9^/L → 19 × 10^9^/L) that resolved spontaneously. A routine ophthalmological examination revealed the presence of ocular albinism. During the first 15 months of life, the patient suffered from various infections, including ethmoiditis, conjunctivitis, stomatitis, cutaneous candidiasis, pneumonia, and several episodes of acute bronchitis. Due to recurrent infections, he was referred to an immunologist. Upon physical examination, the patient had silvery grey hair, pale skin (Figure 1), hepatosplenomegaly, enlarged bilateral cervical and enlarged axillary lymph nodes. Laboratory investigations revealed mild anemia (Hb 10 g/dL), thrombocytopenia (PLT 101 × 10^9^/L), neutropenia (absolute neutrophil count 0.71 × 10^9^/L) in the presence of normal lymphocyte and total white blood cells count (LYMPH 7.08 × 10^9^/L, WBC 8.6 × 10^9^/L). Peripheral blood smear revealed the classic giant azurophilic peroxidase-positive granules in lymphocytes and monocytes. Bone marrow examination also demonstrated similar giant cytoplasmic inclusion in lymphocytes, monocytes, and neutrophil precursors, typically seen in CHS (Figure 2); hence, a diagnosis of CHS was suspected.

### 2.2. Targeted Next-Generation Sequencing

A molecular genetic examination was performed in accordance with the Declaration of Helsinki with written informed consent obtained from each participant and/or their legal representative, as appropriate. Genomic DNA (gDNA) was extracted from peripheral blood mononuclear cells. NGS was performed using a custom target gene panel encompassing 514 genes associated with inborn errors of immunity (Appendix A). Total gDNA was extracted from the patient’s blood sample and sheared by sonication on a Covaris ME220 (USA) to an average fragment size of 250 bp. DNA adapters were added via a ligation-based method using a NebNext Ultra II DNA Library Prep Kit for Illumina (New England Biolabs Inc., Ipswich, MA, USA), according to the manufacturer’s protocol. DNA library preparation was performed by a hybridization-based target enrichment method using SeqCap EZ reagent kits and a custom probe panel manufactured by Roche (Basel, Switzerland), following the manufacturer’s guidelines. DNA libraries were sequenced on an NextSeq platform (Illumina, Inc., San Diego, CA, USA) using 150 bp paired-end mode, according to the standard protocol for this platform. The average depth of target region coverage was 130 reads per bp, 99% of the bases—with target coverage more than 30×. Sequence reads were mapped to the human genome reference sequence (GRCh38/hg38). Single nucleotide variants (SNVs) and short insertions and deletions (INDELs) were detected using a proprietary in-house bioinformatic data analysis pipeline, consistent with international standards. An integrative genomics viewer (IGV) was used for the obtained sequencing data visualization [14]. The SpliceAI neural network was used for preliminary in silico assessment of the impact of the identified genetic variants on splicing [15]. The pathogenicity and clinical significance of the identified variants were assessed in accordance with the criteria of the American College of Medical Genetics/Association for Molecular Pathology (ACMG/AMP) variant interpretation guidelines [16]. Confirmation of the clinically relevant variants and genetic study of proband’s parents and siblings was performed by direct Sanger sequencing on a capillary sequencer Genetic Analyzer 3500XL (Applied Biosystems, Waltham, MA, USA).

### 2.3. cDNA Sequencing

To assess the impact of the identified genetic variants on mRNA splicing, total RNA was isolated from peripheral blood mononuclear cells by phenol-chloroform extraction. The RNA concentration was evaluated using a «Nanodrop One» spectrophotometer (Thermo Fisher Scientific, Waltham, MA, USA). DNAse I (New England Biolabs, Ipswich, MA, USA) was used to remove contaminating genomic DNA from RNA samples, and MINT Reverse Transcriptase (Evrogen, Moscow, Russia) was used for cDNA synthesis. Amplification of the cDNA target regions was performed by PCR using specifically designed primers. The cDNA region, adjacent to the c.10104G>T substitution, was amplified by PCR using forward 5′-CCGTGAAGGTTTTGATTTTGGTG-3′ and reverse 5′-GGAGTCTGCCCGTAGGTTT-3′ primer pair. A cDNA fragment adjacent to the c.10894A>G substitution was amplified by PCR using forward 5′-GCAGCACGCCATCAGAAA-3′ and reverse 5′-AGCTGAATCACACACAGTAGC-3′ primers. The length of the obtained PCR products was evaluated using agarose gel electrophoresis with «DNA Ladder 100+ bp» DNA ladder (Evrogen, Moscow, Russia) and ethidium bromide staining. Sequencing of the obtained cDNA fragments was performed by the Sanger method on a capillary sequencer Genetic Analyzer 3500XL (Applied Biosystems, Waltham, MA, USA).

## 3. Results

The target next-generation sequencing of the patient’s DNA revealed two heterozygous substitutions in *LYST* (NM_000081.4): c.10104G>T p.(Gly3368=) and c.10894A>G p.(Ile3632Val) in exons 44 and 49, respectively. Despite their distance from splice sites, the assessment of these variants by the SpliceAI neural network predicted that both substitutions probably disrupt splicing, leading to the formation of new donor splicing sites in exons 44 and 49, respectively (SpliceAI delta donor gain scores 0.98 and 0.78 for c.10104G>T and c.10894A>G, respectively, Table 1).

**Figure 3:**
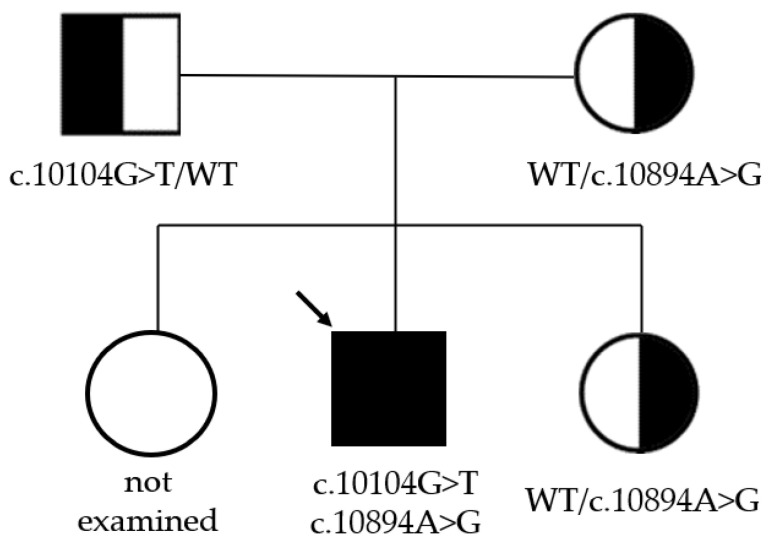
Pedigree of the whole family. The black arrow indicates the proband.

**Figure 4:**
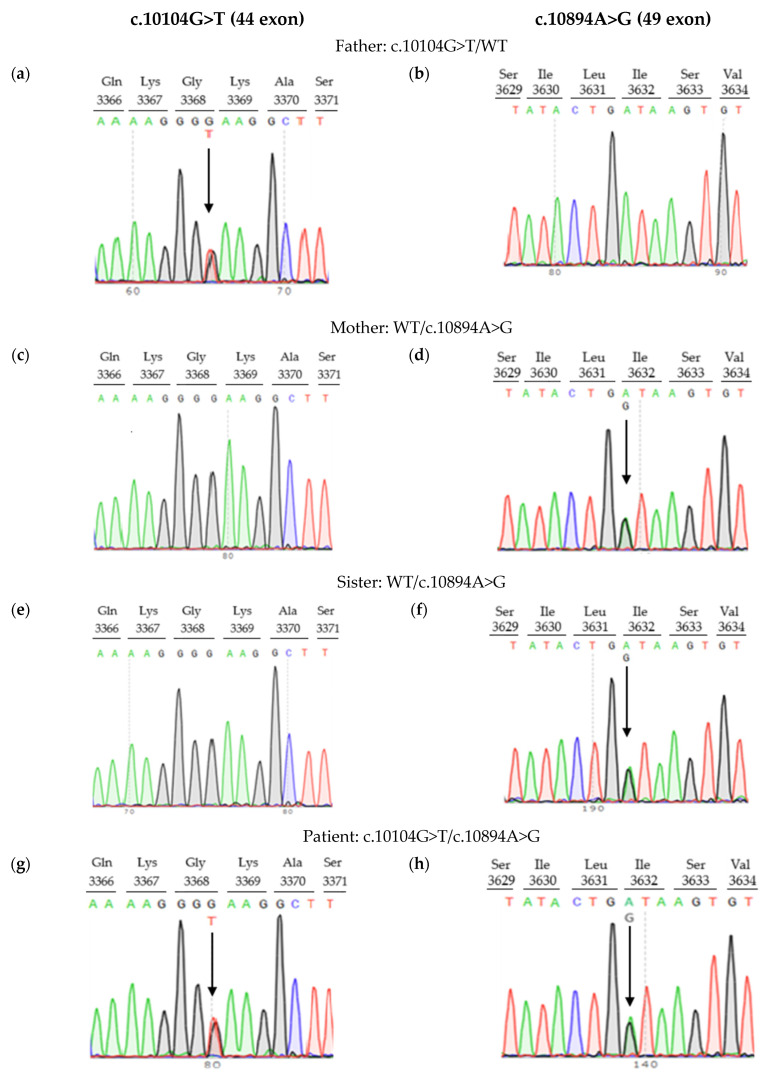
Pedigree information for family with *LYST* variants: (**a**,**b**) father, (**c**,**d**) mother, (**e**,**f**) sister, (**g**,**h**) patient. Arrows indicate nucleotide substitutions.

**Figure 5:**
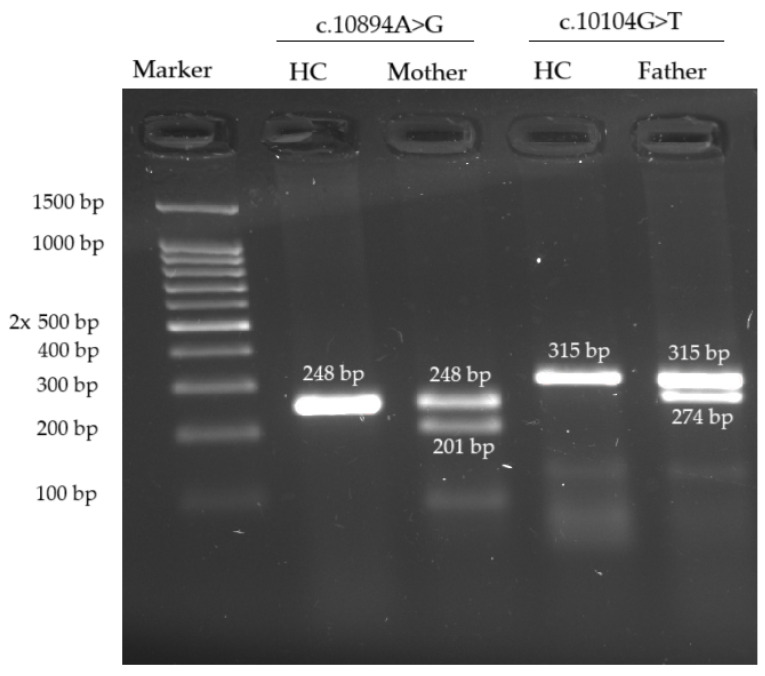
Agarose gel cDNA electrophoresis revealed the presence of an abnormally shortened cDNA fragment in each variant’s case. HC—healthy control. Ethidium bromide staining.

**Figure 6:**
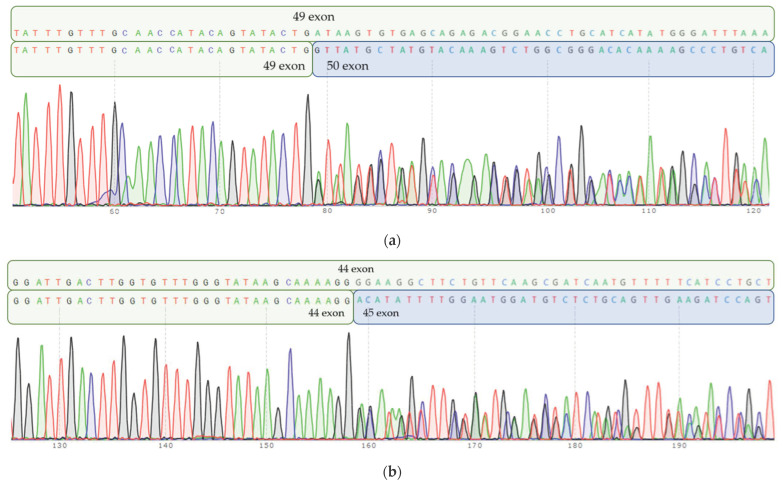
Parents’ cDNA Sanger sequencing. (**a**) Mother’s cDNA Sanger sequencing: heterozygous c.10894A>G substitution in 49 exon leads to inappropriate splicing of RNA and “loss” of 47 b.p. Normal allele sequence (part of 49 exon) is depicted on a top row, and mutant allele (truncated 49 exon, followed by 50 exon) is depicted on the bottom row. (**b**) Fathers’ cDNA Sanger sequencing: heterozygous c.10104G>T substitution in 44 exon results in aberrant RNA splicing and “loss” of 41 b.p. Normal allele sequence (part of 44 exon) is depicted on a top row, mutant allele (truncated 44 exon, followed by 45 exon) is depicted on the bottom row.

**Figure 7:**
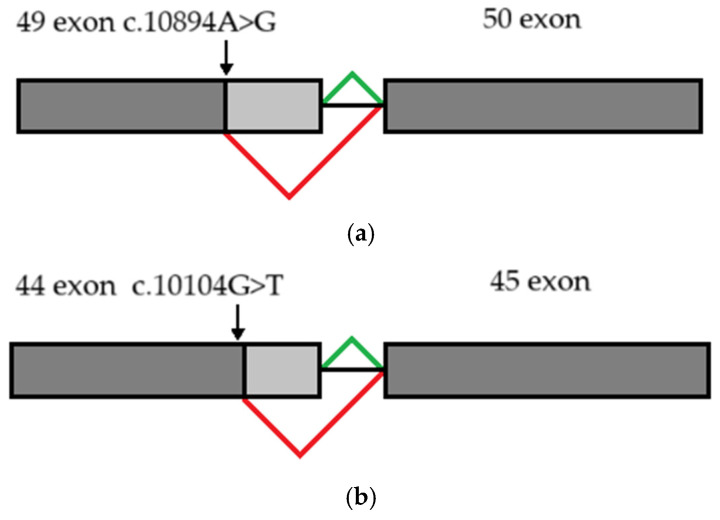
Schematic diagram illustrating abnormal splicing signature: (**a**) due to c.10894A>G substitution (**b**) due to c.10104G>T substitution. Constitutive splicing is depicted by green lines; altered splicing is depicted by red lines.

Neither variant has been previously reported in the literature, and they are not present in population databases (absent from controls in gnomAD and ExAC databases, Table 1). Sanger sequencing of both parents’ DNA demonstrated that the father was a heterozygous carrier of the c.10104G>T substitution, and the mother was a heterozygous carrier of the c.10894A>G substitution. Thus, the transposition of these variants in the patient was confirmed. One of the patient’s sisters also carried the c.10894A>G variant in a heterozygous state; sequencing of the other sister’s DNA was not performed (Figure 3 and Figure 4). In summary, both substitutions were classified as variants of uncertain significance according to the ACMG/AMP guidelines (PM2 and PP3 criteria) [16]. Unfortunately, it was not possible to obtain the patient’s RNA from his peripheral blood mononuclear cells due to a recently performed HSCT. The patient’s parents did not consent to a skin biopsy, so we could not evaluate RNA expression in the patient’s fibroblasts. Therefore, the only way to assess the pathogenicity of these novel variants was to study the parents’ RNA. Agarose gel electrophoresis of both parents’ cDNA fragments resulted in two distinct cDNA bands in each parent’s case, indicating the presence of abnormally shortened cDNA fragments (Figure 5). Sanger sequencing of the parents’ cDNA revealed that c.10104G>T substitution results in a shortening of the 44 exon by 41 bp (NM_000081.4: r.10103_10143del) and c.10894A>G leads to a shortening of the 49 exon by 47 bp (NM_000081.4: r.10894_10940del) (Figure 6 and Figure 7). These data allowed us to confirm an abnormal splicing pattern in both variants’ cases. We suppose that both substitutions lead to a frameshift and formation of premature termination codon, which may disrupt the structure and/or function of the LYST protein. However, the effect of these variants is not fully understood and requires further study at the protein level.

## 4. Discussion

The use of NGS-based technologies is associated with the analysis of a huge amount of genetic data, which can lead to some challenges regarding the interpretation of novel variants. Around 10% of human pathogenic mutations, including variants in coding sequences reported in the Human Gene Mutation Database (HGMD), have been found to affect splicing, and this very likely represents an underestimate [16]. Computational tools such as the SpliceAI neural network make it possible to effectively predict such variants. Sequencing of cDNA helps to assess splicing patterns and confirm the pathogenicity of such genetic variants. To date, about 115 genetic variants associated with Chediak–Higashi syndrome have been reported according to the Human Gene Mutation Database (HGMD), and about 9% of them are described as splice-altering. We performed an analysis of RNA splicing using reverse transcription polymerase chain reaction of RNA obtained from parents’ blood samples and showed that c.10104G>T and c.10894A>G variants disrupt native splicing patterns, resulting in truncation of exon 44 and exon 49, respectively. The obtained data allowed us to reclassify both variants as pathogenic and confirm their causative role in the development of the disease. Unfortunately, we were unable to assess the effect of the identified genetic variants at the protein level in our lab. Thus, the effect of these variants on protein structure and function is not fully understood and requires further investigation.

## Figures and Tables

**Table 1:** Features of the identified genetic variants.

		Variant 1	Variant 2
GRCh38/hg38		chr1:235709130 C>A	chr1:235677526 T>C
GRCh37/hg19		chr1:235872430 C>A	chr1:235840826 T>C
CDS (NM_000081.4)		c.10104G>T	c.10894A>G
Exon		Exon 44	Exon 49
Zygocity		Heterozygous	Heterozygous
SpliceAI donor gain score		0.98	0.78
CADD scores		20.1	26.7
Allele frequency according to population databases	gnomAD v2.1.1	0	0
	gnomAD v3.1.2	0	0
	gnomAD v4.0.0	0	0
	ExAC	0	0

## Data Availability

The original contributions presented in the study are included in the article, further inquiries can be directed to the corresponding author.

## Appendix A

Custom NGS-panel “Inborn errors of immunity” gene list: *A2ML1*, *ABCD4*, *ACD*, *ACP5*, *ACTB*, *ADA*, *ADA2*, *ADAM17*, *ADAMTS3*, *ADAR*, *ADGRE2*, *AGA*, *AICDA*, *AIRE*, *AK2*, *ALG12*, *ALPI*, *ANGPT1*, *AP1S3*, *AP3B1*, *AP3D1*, *ARHGEF1*, *ARPC1B*, *ATM*, *ATP6AP1*, *ATR*, *AURKB*, *B2M*, *BACH2*, *BCL10*, *BCL11B*, *BLM*, *BLNK*, *BLOC1S3*, *BLOC1S6*, *BTK*, *C1QA*, *C1QB*, *C1QC*, *C1R*, *C1S*, *C2*, *C3*, *C3AR1*, *C5*, *C6*, *C7*, *C8A*, *C8B*, *C8G*, *C9*, *CA2*, *CARD11*, *CARD14*, *CARD9*, *CARMIL2*, *CASP10*, *CASP8*, *CBL*, *CBS*, *CCBE1*, *CD19*, *CD247*, *CD27*, *CD28*, *CD3D*, *CD3E*, *CD3G*, *CD40*, *CD40LG*, *CD48*, *CD55*, *CD59*, *CD70*, *CD79A*, *CD79B*, *CD80*, *CD81*, *CD8A*, *CDC42*, *CDCA7*, *CEBPE*, *CFB*, *CFD*, *CFH*, *CFI*, *CFP*, *CFTR*, *CHD7*, *CIB1*, *CIITA*, *CLCN7*, *CLPB*, *COG6*, *COL2A1*, *COL7A1*, *COPA*, *COPG1*, *COPZ1*, *CORO1A*, *CR2*, *CSF2RA*, *CSF2RB*, *CSF3R*, *CTC1*, *CTLA4*, *CTPS1*, *CTSC*, *CXCR4*, *CYBA*, *CYBB*, *CYBC1*, *DBF4*, *DBR1*, *DCLRE1C*, *DDX58*, *DEF6*, *DIAPH1*, *DKC1*, *DNASE1*, *DNASE1L3*, *DNASE2*, *DNMT3B*, *DOCK11*, *DOCK2*, *DOCK8*, *DPAGT1*, *DSG1*, *DTNBP1*, *EDA*, *EDAR*, *EDARADD*, *EFL1*, *EGFR*, *EIF6*, *ELANE*, *ELP1*, *EP300*, *EPCAM*, *EPG5*, *ERBIN*, *EXTL3*, *F12*, *FADD*, *FAM111B*, *FAS*, *FASLG*, *FAT4*, *FBN1*, *FCGR3A*, *FCGR3B*, *FCHO1*, *FECH*, *FERMT1*, *FERMT3*, *FNIP1*, *FOXN1*, *FOXP3*, *G6PC3*, *GATA1*, *GATA2*, *GBA*, *GFI1*, *GIMAP6*, *GINS1*, *GJC2*, *GLA*, *GUCY2C*, *HAVCR2*, *HAX1*, *HELLS*, *HFE*, *HMOX1*, *HPS1*, *HPS3*, *HPS4*, *HPS5*, *HPS6*, *HRAS*, *HSPA1L*, *HYOU1*, *ICOS*, *ICOSLG*, *IFIH1*, *IFNAR1*, *IFNAR2*, *IFNG*, *IFNGR1*, *IFNGR2*, *IGHM*, *IGKC*, *IGLL1*, *IKBKB*, *IKBKG*, *IKZF1*, *IL10*, *IL10RA*, *IL10RB*, *IL11RA*, *IL12B*, *IL12RB1*, *IL12RB2*, *IL17F*, *IL17RA*, *IL17RC*, *IL18BP*, *IL1RN*, *IL21*, *IL21R*, *IL2RA*, *IL2RB*, *IL2RG*, *IL36RN*, *IL6R*, *IL6ST*, *IL7R*, *IRAK4*, *IRF2BP2*, *IRF3*, *IRF4*, *IRF7*, *IRF8*, *IRF9*, *ISG15*, *ITCH*, *ITGA3*, *ITGB2*, *ITGB4*, *ITK*, *ITPKB*, *ITPR3*, *IVD*, *IVNS1ABP*, *JAGN1*, *JAK1*, *JAK3*, *KDM1A*, *KDM6A*, *KMT2A*, *KMT2D*, *KNG1*, *KRAS*, *LACC1*, *LAMA3*, *LAMB3*, *LAMC2*, *LAMTOR2*, *LAT*, *LCK*, *LCP2*, *LIG1*, *LIG4*, *LMBRD1*, *LPIN2*, *LRBA*, *LRP5*, *LRRC8A*, *LYN*, *LYST*, *LZTR1*, *MAGT1*, *MALT1*, *MAN2B1*, *MAP2K1*, *MAP2K2*, *MAP3K14*, *MCM4*, *MEFV*, *MMAA*, *MMAB*, *MMACHC*, *MMADHC*, *MMUT*, *MOGS*, *MPO*, *MRE11*, *MRTFA*, *MS4A1*, *MSN*, *MTHFD1*, *MTRR*, *MVK*, *MYD88*, *MYO5A*, *MYO5B*, *MYOF*, *MYSM1*, *NBAS*, *NBN*, *NCF1*, *NCF2*, *NCF4*, *NCKAP1L*, *NCSTN*, *NF1*, *NF2*, *NFE2L2*, *NFIL3*, *NFKB1*, *NFKB2*, *NFKBIA*, *NHEJ1*, *NHP2*, *NLRC4*, *NLRP1*, *NLRP12*, *NLRP3*, *NLRP6*, *NOD2*, *NOP10*, *NOS2*, *NRAS*, *NSMCE3*, *NUP214*, *OAS1*, *ORAI1*, *OSTM1*, *OTULIN*, *PARN*, *PCCA*, *PCCB*, *PEPD*, *PGM3*, *PIK3CD*, *PIK3CG*, *PIK3R1*, *PLCG2*, *PLEKHM1*, *PLG*, *PLVAP*, *PMM2*, *PNP*, *POGLUT1*, *POLA1*, *POLD1*, *POLD2*, *POLD3*, *POLE*, *POLE2*, *POLR3A*, *POLR3C*, *POLR3F*, *POMP*, *POU2AF1*, *PRDX1*, *PRF1*, *PRKCD*, *PRKDC*, *PSEN1*, *PSENEN*, *PSMA3*, *PSMA5*, *PSMB10*, *PSMB4*, *PSMB8*, *PSMB9*, *PSMC5*, *PSMG2*, *PSTPIP1*, *PTCRA*, *PTEN*, *PTPN11*, *PTPN2*, *PTPRC*, *RAB27A*, *RAC2*, *RAG1*, *RAG2*, *RANBP2*, *RASA2*, *RASGRP1*, *RBCK1*, *RC3H1*, *REL*, *RELA*, *RELB*, *RFX5*, *RFXANK*, *RFXAP*, *RHOG*, *RHOH*, *RIPK1*, *RMRP*, *RNASEH2A*, *RNASEH2B*, *RNASEH2C*, *RNF168*, *RNF31*, *RNU4ATAC*, *RORC*, *RPSA*, *RRAS*, *RRAS2*, *RTEL1*, *SAMD9L*, *SAMHD1*, *SASH3*, *SBDS*, *SC5D*, *SEC61A1*, *SEMA3E*, *SERPINB1*, *SERPING1*, *SH2D1A*, *SH3BP2*, *SH3KBP1*, *SHARPIN*, *SKIV2L*, *SLC29A3*, *SLC35A1*, *SLC35C1*, *SLC37A4*, *SLC39A4*, *SLC39A7*, *SLC39A8*, *SLC46A1*, *SLC7A7*, *SLCO2A1*, *SMARCAL1*, *SMARCD2*, *SMC1A*, *SMC3*, *SMPD1*, *SNORA31*, *SNX10*, *SOCS1*, *SP1*, *SP110*, *SPI1*, *SPINK5*, *SPPL2A*, *SRP54*, *STAT1*, *STAT2*, *STAT3*, *STAT5B*, *STIM1*, *STING1*, *STK4*, *STN1*, *STX11*, *STXBP2*, *STXBP3*, *TAP1*, *TAP2*, *TAPBP*, *TAZ*, *TBK1*, *TBX1*, *TBX21*, *TCF3*, *TCIRG1*, *TCN2*, *TERC*, *TERT*, *TFR2*, *TFRC*, *TGFBR1*, *TGFBR2*, *THBD*, *TICAM1*, *TIMM50*, *TINF2*, *TLR3*, *TLR7*, *TLR8*, *TMC6*, *TMC8*, *TNFAIP3*, *TNFRSF11A*, *TNFRSF13B*, *TNFRSF13C*, *TNFRSF1A*, *TNFRSF4*, *TNFRSF9*, *TNFSF11*, *TNFSF12*, *TNFSF13*, *TOM1*, *TOP2B*, *TPP1*, *TPP2*, *TRAC*, *TRAF3*, *TRAF3IP2*, *TREX1*, *TRIM22*, *TRNT1*, *TSPEAR*, *TTC37*, *TTC7A*, *TYK2*, *UBA1(ex2-3)*, *UNC13D*, *UNC93B1*, *UNG*, *USB1*, *USP18*, *USP43*, *VAV1*, *VPS13B*, *VPS45*, *WAS*, *WDR1*, *WDR44*, *WIPF1*, *WNT2B*, *WNT6*, *WRAP53*, *XIAP*, *XRCC4*, *ZAP70*, *ZBTB24*, *ZNF341* (genes symbols are given according to HUGO Gene Nomenclature Committee).
